# The Role of Mesenchymal Stem Cell Secretome in the Inflammatory Mediators and the Survival Rate of Rat Model of Sepsis

**DOI:** 10.3390/biomedicines11082325

**Published:** 2023-08-21

**Authors:** Mutiara Indah Sari, Nelva Karmila Jusuf, Delfitri Munir, Agung Putra, Tatang Bisri, Syafruddin Ilyas, Farhat Farhat, Adi Muradi Muhar, Muhammad Rusda, Mustafa Mahmud Amin

**Affiliations:** 1 Philosophy Doctor in Medicine Program, Faculty of Medicine, Universitas Sumatera Utara, Medan 20155, Indonesia; m.rusda@usu.ac.id (M.R.); mustafa.mahmud@usu.ac.id (M.M.A.); 2 Department of Dermatology & Venereology, Faculty of Medicine, Universitas Sumatera Utara, Medan 20155, Indonesia; nelva@usu.ac.id; 3 Department of Ear, Nose & Throat, Head & Neck, Faculty of Medicine, Universitas Sumatera Utara, Medan 20155, Indonesia; delfitri_munir@yahoo.com (D.M.); farhat@usu.ac.id (F.F.); 4 Stem Cell and Cancer Research, Faculty of Medicine, Universitas Islam Sultan Agung, Semarang 50112, Indonesia; dr.agungptr@gmail.com; 5 Department of Anesthesiology and Intensive Care, Faculty of Medicine, Universitas Jenderal Achmad Yani, Bandung 40513, Indonesia; tatang.bisri@yahoo.co.id; 6 Department of Biology, Faculty of Mathematics and Natural Sciences, Universitas Sumatera Utara, Medan 20155, Indonesia; syafruddin6@usu.ac.id; 7 Department of Surgery, Faculty of Medicine, Universitas Sumatera Utara, Medan 20155, Indonesia; adibedah@gmail.com

**Keywords:** secretome, NF-κB, TNF-α, IL-10, survival rate, sepsis

## Abstract

In sepsis, simultaneously elevated levels of pro-inflammatory cytokines and interleukin (IL)-10 indicate immune response dysregulation, increasing the mortality of the host. As mesenchymal stem cell (MSC) secretome is known to have immunomodulatory effects, we aim to assess the role of MSC secretome in the inflammatory mediators (NF-κB p65 and p50, TNF-α, IL-10) and the survival rate of a rat model of sepsis. In this study, forty-eight male *Rattus norvegicus* rats were divided into one sham group and three groups with sepsis induction: the control group and the sepsis-induced rat groups treated with 150 μL (T1) and 300 μL (T2) of secretome. The survival rate was observed per 6 h for 48 h and plotted using the Kaplan–Meier method. Compared to the control group, T2 showed a significant decrease in the relative expression of NF-κB and the serum TNF-α level, and a significant increase in the serum IL-10 level. Meanwhile, T1 showed a significant decrease in the serum TNF-α level compared to the control group. The Kaplan–Meier Log Rank test did not show significance in the distribution of survival between T1, T2, and the control group. However, from the 18th to the 36th hour, the survival rate of T2 was lower than the survival rate of the control group and T1, with a noticeable difference between T2 and the control group, as well as T1 at the 36th hour. At the 42nd hour, the survival rate of T2 was the same as the control group and remained lower than T1. In conclusion, MSC secretome regulated the inflammatory mediators in rat model of sepsis, with a dose of 150 μL being more effective.

## 1. Introduction

Sepsis is a medical emergency due to the dysregulation of the body’s immune response to infection [1]. This dysregulation is initiated by the entry of pathogen-associated molecular patterns (PAMPs) such as bacteria, or damage-associated molecular patterns (DAMPs), into the host, which activates nuclear factor kappa B (NF-κB). NF-κB is a central inflammatory mediator and a transcription factor regulating the expression of pro-inflammatory cytokine genes through the activation of intracellular signal transduction pathways. NF-κB activation, especially by pathogenic bacteria, is recognized by Toll-like receptors (TLR)-4. The recognition of lipopolysaccharide (LPS) complexes with the LPS binding protein by TLR-4 leads to the stimulation of one or more redox-dependent kinases so that they phosphorylate IκB [2,3,4]. NF-κB also contributes to the regulation of the survival, activation, and differentiation of various immune cells, such as innate immune cells and inflammatory T cells. A dysregulation in the activation of NF-κB plays a role in the pathogenic process of many inflammatory diseases [5].

Cytokines are small secreted proteins and important mediators in the regulation of immune and inflammatory responses. The main pro-inflammatory cytokines that regulate the early immune response include interleukin (IL)-1α, IL-1β, IL-6, and tumor necrosis factor (TNF)-α. TNF-α is the most studied pro-inflammatory cytokine in sepsis due to its deterministic role in the release of other cytokines. Many studies have found a significant increase in this cytokine in septic patients and animal models [6,7,8,9]. The secretion of these pro-inflammatory cytokines affects the secretion of anti-inflammatory cytokines in order to balance the body defenses [10,11]. In this state, IL-10 will be produced to counteract pro-inflammatory cytokines such as TNF-α [12,13]. However, TNF-α often causes NF-κB activation by itself and forms a positive feedback circuit. Simultaneously, elevated levels of pro-inflammatory cytokines and IL-10 indicate a dysregulation of the inflammatory response. This dysregulation is known as a cytokine storm, which may cause organ damage and secondary infection, leading to increased mortality [14,15,16].

Data obtained in 2016 from studies in several high-income countries found more than 30 million cases of sepsis receiving hospital care, and it was estimated that 5.3 million patients died from sepsis [17]. A global estimation in 2017 found 48.9 million cases of sepsis and 11 million deaths due to sepsis [18]. The rapid administration of the appropriate antimicrobial therapy is key to maximizing the survival rate for sepsis, but the distribution of causative pathogens is constantly changing [19,20]. Moreover, the global increase in antimicrobial-resistant pathogens renders broad-spectrum antimicrobial therapy less effective [20,21,22,23]. Therefore, there is a need for a supportive therapy that can alleviate the cytokine storm and reduce the mortality caused by sepsis.

Mesenchymal stem cells (MSCs) have drawn much interest for their immunomodulatory effects [24]. Many studies have reported that the therapeutic potential of MSCs stems from the paracrine effect of the secreted molecules [25,26]. MSC secretome is known to have immunomodulatory effects, hence its potential in the management of infectious diseases, especially sepsis. The regenerative properties of the secretome also contribute to the repair of organs with pathological conditions associated with inflammation [27]. One study showed that the administration of human embryo MSC secretome significantly increased survival and improved the histopathological score of a mouse model of sepsis. It increased IL-10 without affecting IL-1β and TNF-α [28]. Another study also showed that administering 300 μL of hypoxic-conditioned media from adipose MSC can improve the inflammation in the gastric mucosa of rats [29].

Due to the aforementioned reasons, we aim to assess the role of MSC secretome in inflammatory mediators (NF-κB p65 and p50, TNF-α, IL-10) and the survival rate of rat model of sepsis.

## 2. Materials and Methods

### 2.1. Ethics

The implementation of this research was approved by the Health Research Ethics Committee of Universitas Sumatera Utara (USU) with Letter Number: 541/KEPK/USU/2022. Rats were given food and drink ad libitum and treated according to ethical principles: free from hunger, thirst, discomfort, pain, fear, or stress.

### 2.2. MSC Isolation, Flow Cytometry and Differentiation Analysis

We collected umbilical cords from Wistar rats with a gestational age of 19 days for MSC isolation based on the method used in a past report [30]. The MSCs were cultured in a culture flask containing Dubbelco’s Modified Eagle Medium (DMEM) (Sigma-Aldrich, St. Louis, MO, USA), with 10% Fetal Bovine Serum (FBS) (Gibco™ Invitrogen, Grand Island, NY, USA), 0.25% amphotericin B (Gibco™ Invitrogen, Grand Island, NY, USA) and 1.5% penicillin (100 U/mL)/streptomycin (100 µg/mL) (Gibco™ Invitrogen, Grand Island, NY, USA). The MSCs were incubated at 37 °C with 5% CO_2_ and a medium renewal every three days. At 80% confluency, the MSCs were moved into a new flask. For this study, we used the MSCs from the fourth passage.

Next, we performed a validation test on the cell surface markers using flow cytometry following the instructions of the manufacturer. To stain the membrane antigens, we incubated MSC at 4th passage using CD90.1-PerCP (Clone OX-7), CD29-PE (Clone Ha2/5), CD31-APC (Clone MEC 13.3), and CD45-FITC (Clone OX-1) rat antibodies (BD Bioscience, San Jose, CA, USA) in the dark for 20 min at room temperature. After incubation, we washed the cells twice using 500 μL of phosphate buffer saline (PBS). We then centrifuged the sample at 500× *g* for 5 min. After centrifugation, we then diluted our sample in 300 μL of PBS before analysis. The cells were then investigated using a BD Accuri C6 Plus flow cytometer (BD Bioscience, San Jose, CA, USA). The data were interpreted using BD Accuri C6 Plus software (BD Bioscience, San Jose, CA, USA). Once the MSC culture in the standard medium at 37 °C and 5% CO_2_ reached 95% confluency, the medium was replaced with Rat MesenCult™ adipogenic and osteogenic differentiation basal medium. The medium was supplemented with respective supplement (Stem Cell Technologies, Singapore), 1% L-glutamine (Gibco™ Invitrogen, Grand Island, NY, USA), 1% penicillin (Gibco™ Invitrogen, Grand Island, NY, USA), and 0.25% amphotericin B (Gibco™ Invitrogen, Grand Island, NY, USA), with a renewal every three days. After 21 days of incubation, oil red O and alizarin red (Sigma-Aldrich, St. Louis, MO, USA) staining were performed to identify the adipogenic and osteogenic differentiation, respectively.

### 2.3. Secretome Preparation and Content Analysis

MSCs at 80% confluency were put in the hypoxic chamber. Nitrogen was channeled through the inlet valve, and an oxygen meter was placed in the sensor hole to measure the oxygen concentration within the chamber. Nitrogen was added until the indicator needle showed a concentration of 5% oxygen. The chamber containing the flask was incubated for 24 h at 37 °C.

Upon collection, the cells were centrifuged at 13,000× *g* for ten minutes at 4 °C. The secretome isolation was performed using the Tangential Flow Filtration (TFF) strategy (Formulatrix, Bedford, MA, USA) with molecular weight cut-off categories in accordance with past research [31], using 10–50 kDa 50%, 50–100 kDa 25%, and 100–300 kDa 25% filter cassettes (Formulatrix, Bedford, MA, USA). The secretome was then stored at −80°C before an enzyme-linked immunosorbent assay (ELISA) was performed to analyze the contents, in accordance with the manufacturer’s instructions (Invitrogen, Carlsbad, CA, USA).

### 2.4. Examination of Microbial Contamination in Feces

The most probable number (MPN) method was used to estimate the concentration of microorganisms in feces by replicating growth broth in a tenfold dilution of 1 mL of sample. Each dilution was inoculated into a triple tube of broth culture for incubation. The nutrient broth was meant to support the growth of organisms and become murky. The assessment was performed based on the MPN table from the Food and Drug Administration (FDA) bacterial analysis manual [32].

### 2.5. Sample Size dan Animal Model Procedure

Male *Rattus norvegicus* rats aged 10–12 weeks and weighing 200–300 g were obtained from the Animal House of Stem Cell Research Center (SCCR) of Universitas Islam Sultan Agung (Unissula) Faculty of Medicine, Semarang. Rats were kept in different cages at room temperature (37 °C) and given food and drink in moderation, as well as 12 h of light intensity. Before sepsis induction, rats were reared for 7 days to ensure that no other diseases were present.

Based on the power analysis formula, with the possibility of dropping out taken into account [33,34], the sample size for this study was 48, with each group consisting of 12 rats. The rats were divided into four groups: the sham group, the sepsis-induced control group, and the sepsis-induced rat groups, which were given 150 μL (T1) and 300 μL (T2) of secretome.

To obtain the animal model of sepsis, feces was obtained from the cecum of fresh donor rats and then mixed with sterile saline solution at a concentration of 90 mg/mL to produce a fecal suspension. The suspension was injected intraperitoneally at a dose of 1 g/kg body weight [35] to the lower-right quadrant of the rat abdomen using a 21-gauge needle. To avoid trauma from needle insertion, the abdominal wall was lifted with tweezers [36]. Four hours after the sepsis induction, the antibiotic imipenem at a dose of 25 mg/kg body weight in 150 µL of physiological saline was administered intraperitoneally to the septic rat groups for ethical considerations [37,38]. Secretome was administered to T1 and T2 four hours after sepsis induction [29,35,36].

### 2.6. Survival Rate

The sepsis survival rate was observed in each group of rats per 6 h for 48 h [31]. Observations were analyzed as a percentage based on the number of dead (non-survivor) and surviving (survivor) septic rats.

### 2.7. Biomarker Measurement

After 48 h of survival rate observation, all rats were terminated by administering anesthesia using 80 mg/kgBW of ketamine and 4 mg/kgBW of xylazine via the intramuscular route. Blood was taken to measure the relative expression of NF-κB p65 and p50 and the serum levels of TNF-α and IL-10.

### 2.8. Real-Time Polymerase Chain Reaction (RT-PCR) of Gene Expression

To measure the relative expression of NF-κB p65 and p50, we used lysing solution (BD Bioscience, San Jose, CA, USA) to isolate the peripheral blood mononuclear cells (PBMCs) from the rat blood. The effect of secretome on the relative expression of NF-κB was measured via RT-PCR (Tri Reagent, Merck, Readington, NJ, USA). RNA was isolated using ready-to-use kit according to the instructions of the manufacturer. For the experiment, we used the following primers: NF-κB p65 forward 5′-AACACTGCCGAGCTCAAGAT-3′ and reverse 5′-CATCGGCTTGAGAAAAGGAG-3′ primer; NF-κB p50 forward 5′-AGAGCAACCGAAACAGAGAGG-3′ and reverse 5′-TTTGCAGGCCCCACATAGTT-3′ primer; β-actin forward 5′-GTCAGGTCATCACTATCGGCAAT-3′ and reverse AGAGGTCTTTACGGATGTCAACGT primer [39,40].

### 2.9. ELISA of Serum Cytokine Levels

Blood was taken from the rats’ orbital and centrifuged at 2000× *g* for 20 min. The serum was then taken and frozen at −20 °C. Serum levels of TNF-α and IL-10 were analyzed following the instructions of the ELISA kit (Bio-Rad, Hercules, CA, USA).

### 2.10. Statistical Analysis

The data concerning NF-κB expression and TNF-α and IL-10 levels were presented as means. Data with a normal distribution were analyzed using one way ANOVA, and an LSD post hoc test was performed on the significant data. Meanwhile, data without normal distribution were analyzed using Kruskal–Wallis analysis and a Mann–Whitney post hoc test was performed on the significant data. *p* < 0.05 indicates significance. The sepsis survival data were plotted using the Kaplan–Meier method and analyzed using the log rank test.

## 3. Results

### 3.1. MSC Characterization and Secretome

**Figure 1:**
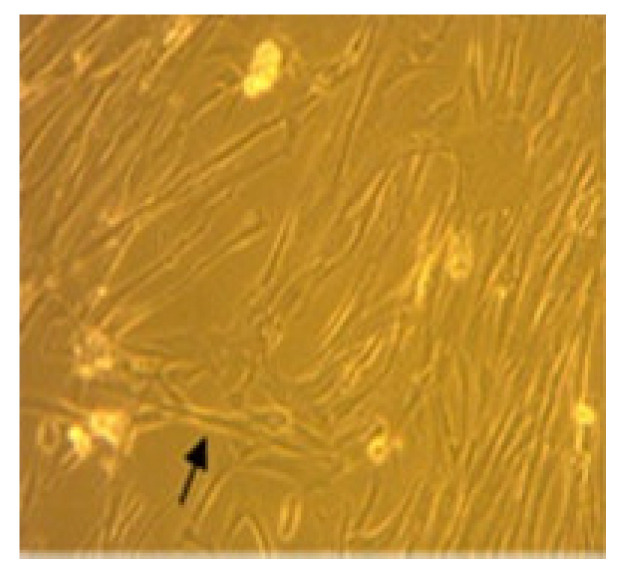
MSC isolation with 80% confluence. Arrow indicates the spindle-like morphology.

The cell specimen attached to the bottom of the flask showed spindle-like cell morphology under microscopic examination (Figure 1).

**Figure 2:**
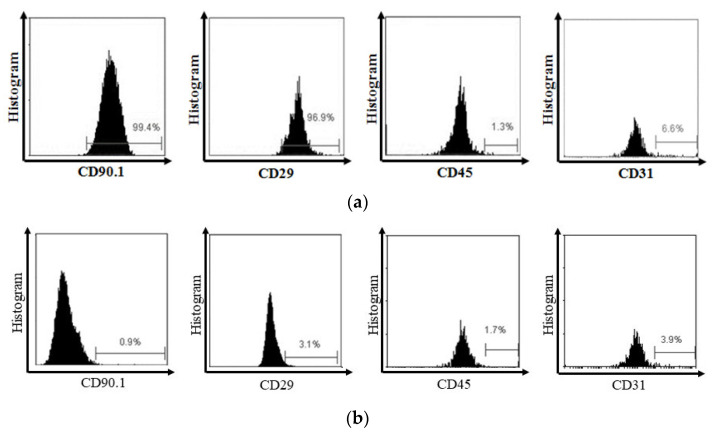
(**a**) Flow cytometry analysis showed that MSC expressed CD90.1 and CD29, and did not express CD45 and CD31. (**b**) On the other side, the isotype showed the negative expression of CD90.1, CD29, CD45, and CD31.

MSC flow cytometry analysis showed a high expression of CD90.1 and CD29 MSC markers and a low expression of CD45 and CD31 hematopoietic markers (Figure 2).

**Figure 3:**
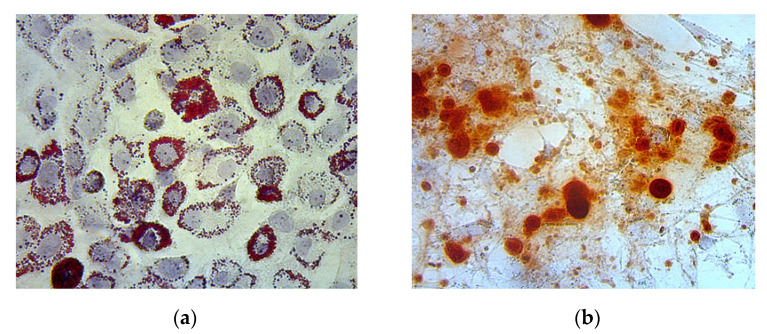
(**a**) Adipogenic differentiation is represented by lipid deposition on the MSC culture, indicated by the color red; (**b**) Osteogenic differentiation is represented by calcium deposition on the MSC culture, indicated by the color red.

Oil red O and alizarin red staining of the MSC culture showed lipid and calcium deposition, indicating adipogenic and osteogenic differentiation, respectively (Figure 3).

### 3.2. Secretome Content Analysis

In this study, the normoxic MSC secretome consisted of IL-10 at 150.61 ± 15.32 pg/mL, transforming growth factor (TGF)-β at 73.53 ± 7.15 pg/mL, and TNF-α at 3.55 ± 0.59 pg/mL. On the other hand, the hypoxic MSC secretome consisted of IL-10 at 269.57 ± 38.39 pg/mL, TGF-β at 138.15 ± 6.12 pg/mL, and TNF-α at 4.93 ± 1.04 pg/mL (Table 1).

### 3.3. Examination of Microbial Contamination in Feces

**Figure 4:**
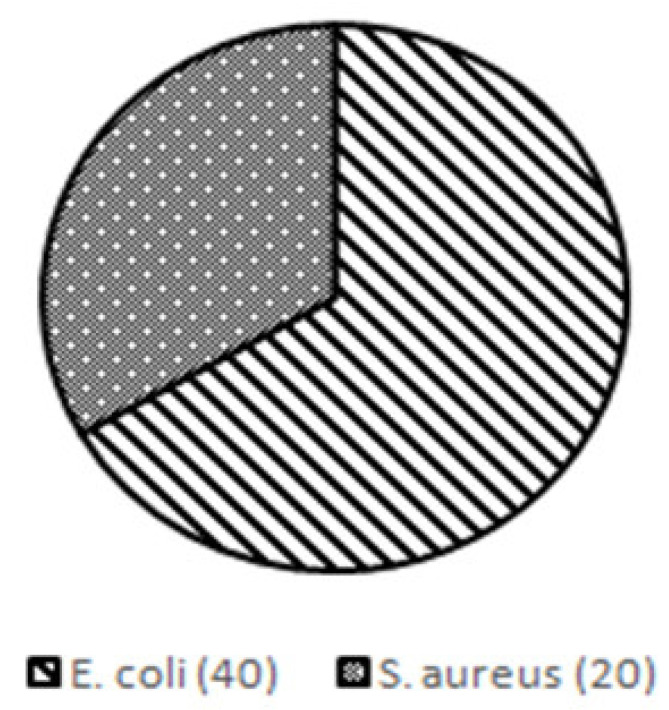
Results of microbial contamination test in rat feces used for sepsis induction.

The microbial contamination test found 40 MPN/gr (67%) of *Escherichia coli* (*E. coli*) and 20 MPN/gr (33%) of *Staphylococcus aureus* (*S. aureus*) in the rat feces (Figure 4).

### 3.4. Effect of Secretome on NF-κB

**Figure 5:**
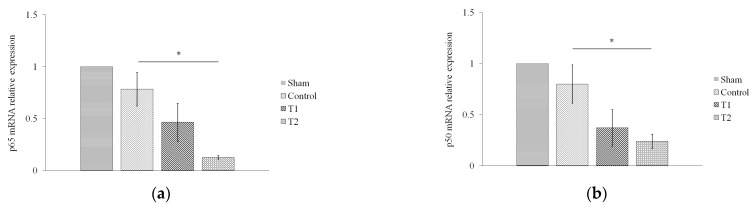
Effect of MSC secretome on the relative expression of NF-κB (**a**) p65 and (**b**) p50 in a rat model of sepsis. Rats were given secretome with different doses (150 and 300 μL). * indicates significance at *p* < 0.05.

To determine the effect of secretome on immune system recovery during sepsis, we assessed the relative expression of NF-κB p65 (Figure 5a) and p50 (Figure 5b) via RT-PCR. The results showed a significant difference between the effect of secretome administration and the decrease in the relative expression of p65 and p50 mRNA in T2 compared to the control group (*p* = 0.049; *p* = 0.042), but not between T1 and the control group (*p* = 1.000; *p* = 0.357).

### 3.5. Effect of Secretome on Pro-Inflammatory and Anti-Inflammatory Secretome

**Figure 6:**
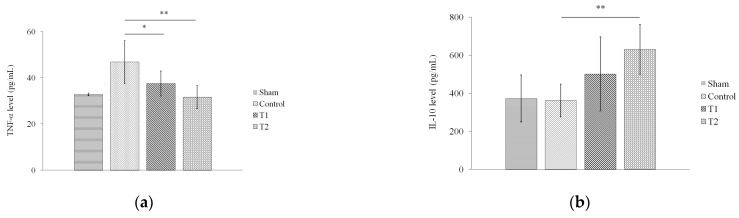
Effect of MSC secretome on the levels of (**a**) TNF-α and (**b**) IL-10 in a rat model of sepsis. Rats were given secretome with different doses (150 and 300 μL). * indicates significance at *p* < 0.05; ** indicates significance at *p* < 0.01.

Next, we assessed the levels of TNF-α and IL-10 in rat serum via ELISA. The results showed a significant decrease in the serum TNF-α level in T1 and T2 compared to the control group, with *p* < 0.05 (*p* = 0.024; *p* = 0.001, Figure 6a). On the other hand, the results also showed that there was a significant increase in the serum IL-10 level in T2 compared to the control group (*p* = 0.008), but not between T1 and the control group (*p* = 0.132, Figure 6b).

### 3.6. Kaplan-Meier Analysis of Survival

**Figure 7:**
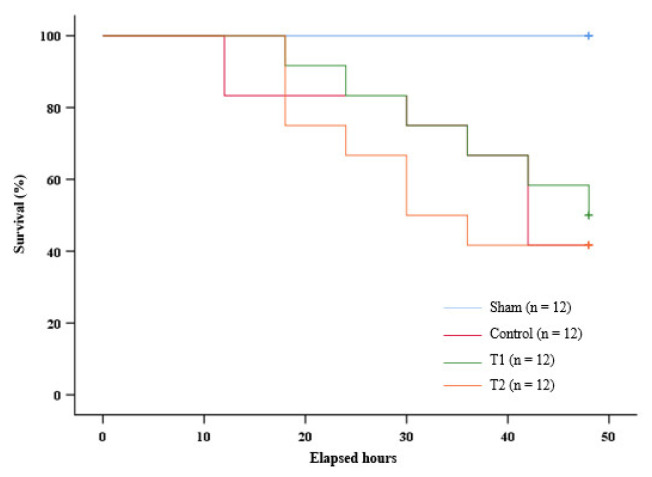
Kaplan–Meier survival analysis graph.

The Kaplan–Meier Log Rank test did not show significance (*p* = 0.770) in the distribution of survival between the control group, T1, and T2. As shown by the graph (Figure 7), all the rats in the sham group survived throughout the observation. At the 12th hour of the observation, the survival rate of the control group was lower than the survival rate of T1 and T2. However, at the 18th hour, the survival rate of T2 was lower than the survival rate of the control group and T1. This trend persisted to the 36th hour of the observation, with a noticeable difference between T2 and the control group, as well as T1 at the said hour. At the 42nd hour, the survival rate of T2 was the same as the control group and remained lower than T1. These results indicate that the administration of MSC secretome at 150 μL in T1 increased the survival rate in septic rats.

## 4. Discussion

Sepsis is a condition consisting of an impaired host response to infection and can occur in infections with bacteria that produce pathogenic products. *S. aureus* and *Streptococcus pneumoniae* are the most common Gram-positive bacteria found in blood cultures of sepsis patients, while *E. coli*, *Klebsiella* sp., and *Pseudomonas aeruginosa* are predominant among Gram-negative bacteria [41,42]. In this study, an experimental animal model of sepsis was obtained via the fecal-induced peritonitis (FIP) method in *Rattus norvegicus* rats. Although cecal ligation and puncture (CLP) is a gold standard for sepsis induction in a murine model, FIP shows less variability among the individual animals. FIP is minimally invasive and does not result in surgical trauma and tissue ischemia, unlike CLP [43,44].

The feces used in this study were first subjected to microbiological tests to determine the content of bacterial contamination. We calculated the MPN of the microorganism for microbiological tests. We used the MPN method because it can detect the fermentative nature of coliforms in samples. The MPN also showed better sensitivity than the colony-forming unit (CFU) method. A previous study revealed that performing the *E. coli* calculation using the MPN resulted in more preferable concentrations than the CFU method [45]. In this study, we reported that the microbial fecal contamination of *E. coli* was 40 MPN/gr (67%) and that of *S. aureus* was 20 MPN/gr (33%). The administration of Gram-positive (*Streptococcus pneumoniae* and *S. aureus*) or Gram-negative (*E. coli*, *Bacteroides fragilis*, *Klebsiella pneumonia*, *Acinetobacter baumannii*, and *Pseudomonas aeruginosa*) bacteria is an easily reproducible and less invasive method of inducing sepsis, suitable for activating the immune response to specific pathogens without surgery. *E. coli* is commonly used among Gram-negative bacteria, while Staphylococcus and Pseudomonas are commonly used among Gram-positive bacteria. As commensal bacteria living in the digestive tract of rats, *S. aureus* and *E. coli* can be found in rat feces [46,47,48].

In this study, MSC secretome was prepared from the umbilical cord of pregnant rats in several stages. We applied controlled hypoxia to MSCs to obtain a higher number of anti-inflammatory molecules on the MSC secretome. Under hypoxic conditions, MSCs exhibit increased expressions of anti-inflammatory molecules and growth factors. This phenomenon is attributed to the controlled stress caused by oxygen deficiency, which can affect the activation of various hypoxia-dependent pathways, including HIF-1a and STAT-3 [49,50]. An analysis of the hypoxic MSC secretome contents showed a higher amount of IL-10 and TGF-β compared to normoxic MSC secretome. On the other hand, both normoxic and hypoxic MSC secretome have low levels of TNF-α, indicating low levels of pro-inflammatory mediators in MSC secretome. MSC secretome contains various cytokines, one of which is IL-10. IL-10 has anti-inflammatory effects and is capable of inhibiting the NF-κB pathway. IL-10 reduces the degradation of IκB-α and the translocation of p65 to the nucleus, suppressing pro-inflammatory activity [1,11]. IL-10 can act as a regulator of T helper 2 (Th2) cells and as an immunomodulator directly on CD4+ T cells to stimulate Th2 polarization [51]. Th2 produces IL-4 and leads to increased IL-10 secretion through a positive feedback loop by stimulating the polarization of M1 to M2 macrophages [52,53].

In addition to cytokines, MSC secretome contains growth factors, including TGF-β. The name TGF-β originates from its ability to cause normal cell transformation. However, TGF-β also functions outside of cellular transformation. It can induce the expression of SMAD7, which inhibits the activation of TGF-β-activated kinase 1 (TAK1). This inhibits the phosphorylation of IκB-kinase α (IKKα) and thus prevents NF-κB activation [54,55].

This is in line with our findings, which showed a significant decrease in the mRNA expression of NF-κB (p65 and p50) upon the administration of MSC secretome in septic rats. The administration of 300 μL of secretome significantly reduced the mRNA expression of p65 and p50 compared to the control group. The IL-10 within MSC secretome exerts anti-inflammatory effects through the IκB-α degradation pathway and p65 translocation, as well as the activation of T-regulatory cells (Treg), while TGF-β inhibits the NF-κB pathway. These will reduce the production of pro-inflammatory cytokines such as TNF-α. TNF-α is a major pro-inflammatory molecule first identified in 1975 and known to exert inflammatory effects. TNF-α is often the main target in the treatment of various inflammatory diseases and plays a role in infectious disease and sepsis pathophysiology [56,57]. In this study, we found a significant decrease in the serum TNF-α level in the septic rat group after MSC secretome administration at a dose of 150 µL and 300 µL compared to the control group.

In an inflammatory environment, TGF-β also plays a role in the polarization of M1 to M2 macrophages via the Akt/FoxO1 pathway. FoxO1 is one of the downstream targets of the Akt pathway and has been reported to regulate macrophage polarization through phosphorylation. TGF-β increases the phosphorylation of FoxO1, which causes the translocation of the biomolecule and ultimately M2 polarization. M2 macrophages produce anti-inflammatory cytokines such as IL-10 [58,59,60]. In line with this, we also found an increase in the IL-10 level in the serum of the septic rat group after MSC secretome administration compared to the control group, with significance found in the rat group given 300 μL of MSC. IL-10 is a homodimeric cytokine produced by immune cells such as natural killer (NK) cells, macrophages, monocytes, and B and T lymphocytes. IL-10 can suppress the secretion of pro-inflammatory cytokines, such as TNF-α [11,12].

However, studies have shown that extreme levels of these biomarkers are associated with poor outcomes due to immunoparalysis. Previous studies have demonstrated that knock-out studies of mice deficient in components of the NF-κB system had an increased susceptibility to infection, whereas overactivation resulted in multiorgan inflammation and failure [24,61,62,63]. Moreover, extremely low levels of TNF-α reduce the ability to recruit immune cells through chemokine induction and the supraregulation of adhesion molecules. Overly low pro-inflammatory cytokine levels can interfere with the function of these cytokines in controlling mycobacterial infections [8,64]. Meanwhile, IL-10 could increase Treg proliferation, which contributes to immunoparalysis through the anergy of T cell lymphocytes, which is the failure of cells to proliferate, secrete cytokines, and respond to antigens. This prevents the septic individual from setting up an effective immune response to secondary infections, increasing the severity of sepsis and leading to a poor prognosis [54,65,66,67].

Although the Kaplan–Meier Log Rank test did not show significance in the distribution of survival, the survival rate of the rat group treated with 300 µL of MSC secretome remained consistently lower than the survival rate of the control group and the rat group treated with 150 µL of MSC secretome from the 18th to the 36th hour of the observation. There was also a noticeable difference at the 36th hour of the observation. It is worth noting that the rat group given 300 µL of MSC secretome showed a significant decrease in the mRNA expression of NF-κB and serum TNF-α levels compared to the control group. The serum IL-10 levels of this rat group showed a significant increase compared to the control group. Taken together, this suggests that a secretome at a higher dose caused immunoparalysis to occur earlier in septic rats.

On the other hand, the septic rats treated with 150 µL of MSC secretome showed a stabler survival rate compared to the control group and the rat group treated with 300 µL of MSC secretome. In particular, the rat group treated with 150 µL of MSC had a higher survival rate compared to the control group at the 12th, 18th, and 42nd hours. At the end of the observation period, this rat group also had a higher survival rate compared to the control group and the rat group treated with 300 µL of MSC secretome. Interestingly, the rat group given 150 µL of MSC secretome showed only a significant decrease in the serum TNF-α level when compared to the control group. Therefore, the balance in the immune response is crucial in treating sepsis.

Another interesting observation that we found was that, on the 12th hour of the observation, the control group showed a lower survival rate compared to both septic rat groups with MSC secretome administration. These data suggest that the survival rate of the septic rats treated with MSC secretome at a higher dose than the control group is at a point in which cytokine imbalance or immune system dysregulation has not occurred. It is suspected that the dysregulation caused by an alteration in the T cell compartment is associated with high mortality caused by sepsis [68,69]. Past studies have also noted the importance of the timing of MSC secretome administration in sepsis, as it plays an important role in determining the positive or negative effects of IL-10. In the early phase of sepsis, IL-10 provides a protective effect. Administering IL-10 within hours of the onset of sepsis suppresses TNF-α secretion and prolongs the therapeutic potential of rescue surgery [13,70,71]. However, over-administration of the IL-10 contained in MSC secretome could play a role in immunosuppression in the later phases of sepsis, which correlates with increased sepsis severity and poor prognosis [11,12,13,72,73,74].

The strength of this study is that it observes the effect of MSC secretome administration on the biomolecular changes and survival in a rat model of sepsis, as well as how the dose of secretome impacts the survival rate. However, this study also has its limitations. It does not assess the effect of secretome on organ degeneration, such as changes in the histopathological features of the lungs or kidneys. It does not examine the improvement in organ function either, such as assessing kidney function through urea-creatinine levels. We also did not assess the mRNA or intracellular protein expression of IL-10 and TNF-α in PBMCs of septic rats. By measuring only serum levels, it may introduce bias regarding whether IL-10 and TNF-α belong to PBMCs or MSC secretome. We hope to address this limitation in our future study.

## 5. Conclusions

The administration of MSC secretome gave an immunoregulatory effect in the form of a decreased expression of NF-κB p65 and p50, as well as a decrease in the serum TNF-α level, and an increased serum IL-10 level in rat model of sepsis. However, rats given MSC secretome at a dose of 150 μL showed a stabler survival rate compared to the rats given MSC secretome at a dose of 300 μL. This could be attributed to the paralytic effect due to excessive immunosuppression at higher doses. Therefore, we recommend a dose of 150 μL in treating sepsis to avoid inducing an immunoparalytic state. Further studies of this therapy from a clinical perspective assessment are recommended for a better evaluation of the effectiveness of MSC secretome.

## Figures and Tables

**Table 1:** Analysis of soluble molecules from normoxic and hypoxic MSC secretome. The analysis was employed using ELISA.

Molecules	Normoxic MSC Secretome Value ± SE (pg/mL)	Hypoxic MSC Secretome Value ± SE (pg/mL)
IL-10	150.61 ± 15.32	269.57 ± 38.39 ***
TGF-β	73.53 ± 7.15	138.15 ± 6.12 ***
TNF-α	3.55 ± 0.59	4.93 ± 1.04

*** indicates significance.

## Data Availability

The data presented in this study are available upon request from the corresponding author.
